# Manual measurement of SD-OCT images of hyperreflective retinal layers in acute retinal vessel occlusion is reliable, repeatable and reproducible

**DOI:** 10.1038/s41598-025-20086-7

**Published:** 2025-10-03

**Authors:** Egbert Matthé, Dierk Wittig, Matthias Kuhn, Peggy Eulitz, Katharina Schoen, Dirk Sandner, Olga Furashova

**Affiliations:** 1 Medizinisches Versorgungszentrum Neustadt in Sachsen, Böhmische Straße 4, 01844 Neustadt in Sachsen, Germany; 2 Klinik und Poliklinik für Augenheilkunde, Carl Gustav Carus Faculty of Medicine, Technische Universität Dresden, Fetscherstraße 74, 01307 Dresden, Germany; 3 Institute for Medical Informatics and Biometry (IMB), Carl Gustav Carus Faculty of Medicine, Technische Universität Dresden, Fetscherstr. 74, 01307 Dresden, Germany; 4 Ophthalmologists Dres. Bau und Bottesi, Bautzner Str. 66, 01099 Dresden, Germany; 5 Ophthalmologists Dres. Wilke, Bayreuther Straße 30, 01187 Dresden, Germany; 6 Department of Ophthalmology, Klinikum Chemnitz gGmbH, Flemmingstraße 2, 09116 Chemnitz, Germany

**Keywords:** Retinal artery occlusion, Retinal vein occlusion, OCT images, Reflectivity changes, Retinal ischemia, Biomarkers, Medical research

## Abstract

To investigate the repeatability and reproducibility of manual measurements of the reflectivity of retinal layers in healthy eyes and in eyes with acute retinal vessel occlusion via spectral-domain optical coherence tomography (SD-OCT). To reflect the full spectrum of different reflectivities seen in OCT images, for each of the following conditions, exactly one patient with a healthy and an affected eye was selected: non-ischemic central retinal vein occlusion (CRVO), ischemic CRVO, incomplete, subtotal and total central retinal artery occlusion (CRAO). For all the patients, OCT images of the healthy and affected eyes were used. Each image was presented 10 times in a randomly changing sequence to all examiners (one experienced, two nonexperienced). The obtained values were analyzed via two statistical methods: Gage Repeatability & Reproducibility (Gage R&R, a measurement system analysis) and Lin’s concordance correlation coefficient (Lin’s CCC). The total gage R&R values were as follows: vitreous body (VB) 1.11%, innermost retinal layer (IMRL) 0.94%, middle retinal layer (MRL) 4.94%, inner retinal layer (IRL) 2.56% and outer retinal layer (ORL) 3.77%. The repeatability values were 0.79, 0.62, 2.80, 1.42 And 2.60, respectively. The reproducibility values were 0.32, 0.33, 2.41, 1.14 and 1.16, respectively. Our previously described method of manual measurement of SD-OCT images of retinal layers determining the level of reflectivity (and hence ischemia) in the retina without any invasive examination is reliable, repeatable, reproducible and feasible for clinical use. Registry: ClinicalTrials.gov, Protocol ID: EK 417,102,016, TRN: NCT03061526, Registration date: 19 February 2017.

## Introduction

Retinal vessel occlusions are very common, but despite a vast and growing body of knowledge, their management still remains challenging^1–3^.

Retinal artery occlusion (RAO) leads to immediate severe ischemia of the retina and causes sudden painless loss of vision^4^. At initial presentation, the degree of vision loss differs from no light perception to 20/25 and is strongly dependent on the type (central or branch retinal artery occlusion) and involvement of fovea or papillo-macular bundle. 90% of patients present with visual acuity of 20/400 or less in cases of central retinal artery occlusion^5^. The visual outcome has been shown to be very poor, with very little visual improvement.

Several studies have shown stage-dependent visual outcomes of retinal artery occlusion after treatment, as well as correlations between baseline and final visual acuity depending on the stage of ischemia^6–8^. Therefore, a convenient and easy measurement method as well as a grading system for acute ischemia in retinal artery occlusion are clinically important and have been introduced by our study group in past publications. We showed that retinal ischemia in acute retinal artery occlusion results in hyperreflectivity of the inner retinal layers. The degree of this reflectivity change is different and depending on the disease grade which might therefore be used to assess the degree of acute ischemic damage to the retina^9,10^.

The course of retinal vein occlusions (RVOs) is strongly dependent on the absence or presence of retinal ischemia. While the non-ischemic RVO type usually results in visual recovery of up to 100%, the ischemic RVO type often results in very poor visual outcomes with severe complications up to painful neovascular glaucoma with consecutive evisceration or enucleation^11^. Given the importance of retinal ischemia, many studies have been searching for reliable markers of it in recent years. In the Central Vein Occlusion Study (CVOS), fluorescein angiography (FA) was used to distinguish ischemic from non-ischemic RVO^12,13^: the loss of capillary fluorescence (loss or closure of retinal capillaries) defines ischemic conditions. Currently, FA remains the gold standard for providing detailed information about non-perfused areas of the retina. However, the rule of at least “10 disc areas of capillary non-perfusion” for defining clinically relevant ischemic central retinal vein occlusion (CRVO) might be misleading: as it does not consider the localization of the ischemic areas in relation to the fovea, it cannot always correlate with the visual outcome^1,14^. Furthermore, this definition does not and cannot take into account the objective ischemic situation in the retina itself, which might be independent of capillary loss or precedes this loss. Finally, FA is an invasive diagnostic tool with possible adverse reactions and a subjective assessment of non-perfused areas.

The relatively new method of optical coherence tomography angiography (OCTA) has also been widely used on this topic. By taking a series of pictures in a very short time frame, changing structures, which are basically moving blood cells, can be identified and combined with the three-dimensional information of the retinal structure. Therefore, in contrast to FA, which lacks three-dimensional information, OCTA can analyze several capillary layers and their pathologic situation in retinal vessel occlusions. In recent years, several of the main problems associated with this method, such as its limited angle of view and mandatory high level of experience in data interpretation, have been overcome, and a consensus has been established for acquiring and interpreting OCTA images^15^. The concept of capillary dropout seen in FA could be refined by identifying the deep capillary plexus as the main problem in CRVO^16^both for visual acuity and for macular edema. Furthermore, a correlation between central and peripheral capillary dropout can be established^17^.

The general downsides of this method are that it is still less commonly available than OCT or FA and that interpreting the images is more time consuming and requires more experience. With respect to detecting ischemia, another downside is that, like FA, it cannot detect (intra)cellular ischemia and hence is not able to quantify acute RAO cases, where there is no capillary dropout.

Because this method was not available when our study started, we did not include OCTA data in our work.

In any retinal occlusive disorder – arterial or venous – reliable assessment of retinal ischemia in the acute stage is mandatory for making correct decisions about therapeutic strategies, the prognosis of visual acuity, and the disease course^18–20^. However, most of the parameters used to date, such as visual acuity, relative afferent pupillary defects, and perimetry, remain subjective and/or represent only indirect hints of the possible extent of retinal ischemia in acute retinal vessel occlusion. Electrophysiological investigations help determine the extent of ischemic retinal damage, but these technologies are not easily available in most ophthalmological offices and often refer to the retina as a whole and only to a lesser extent (multifocal electroretinography) to specific retinal areas.

Optical coherence tomography (OCT) is a non-invasive technology that provides in vivo high-resolution cross-sectional images of the retina. On the basis of OCT images, microscopic damage to the retina in different layers can be identified and observed over time^21–25^. In the past, our measurement of reflectivity (or optical intensity or optical density) has been used to establish a reliable grading system of the severity of retinal artery occlusion as well as an estimation of the ischemic situation of retinal vein occlusions^9,10,26^. However, to date, this method involves manual work and is therefore prone to subjective influences and inaccuracies, which could hamper reliability and reproducibility. As there is no objective method offered by industry (preferably, one based on the OCT’s raw data would be needed) we examined the reliability and reproducibility of the aforementioned method.

## Results

### Demographics, original OCT images, mode of presentation

Table 1 shows the demographics of the selected patients.

**Table 1:** Demographics of the selected patients.

No	Sex	Age	Diagnosis	Grade	Affected eye
1	male	51	CRAO	incomplete	Left eye
2	male	56	CRAO	subtotal	Right eye
3	female	81	CRAO	total	Left eye
4	female	58	CRVO	non-ischemic	Left eye
5	male	56	CRVO	ischemic	Right

Abbreviations used: *CRAO* central retinal artery occlusion, *CRVO* central retinal vein occlusion.

We randomly selected patients from a larger cohort used in the previous work. Two male patients and one female patient with CRAO were included. For incomplete CRAO, a 51-year-old male whose left eye was affected was chosen. For subtotal CRAO, a male at the age of 56 years with the right eye affected was included, and for total CRAO, a female at the age of 81 years and the left eye affected was included. Patients with BRAO were not selected.

For venous occlusions, two patients with CRVO and no BRVO were chosen randomly: a 58-year-old female with non-ischemic CRVO on her left eye and a 56-year-old male with ischemic CRVO on his right eye.

**Fig. 1:**
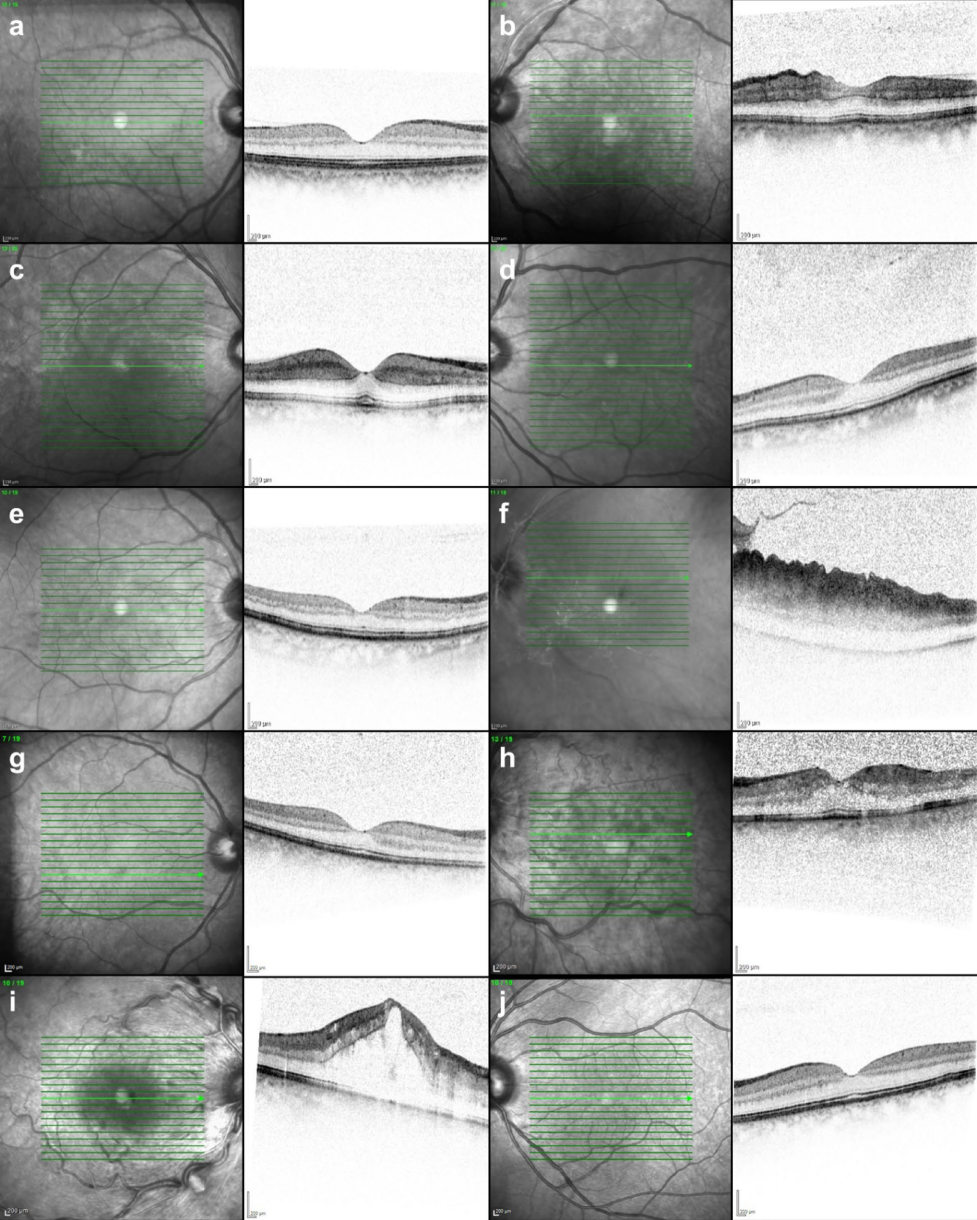
OCT images used for the statistical evaluation of both eyes of each of the five patients. a + b: patient 1; a: right eye, healthy; b: left eye, incomplete CRAO. c + d: patient 2; a: right eye, subtotal CRAO; b: left eye, healthy. e + f: patient 3; a: right eye, healthy; b: left eye, total CRAO. g + h: patient 4; a: right eye, healthy; b: left eye, non-ischemic CRVO. i + j: Patient 5; a: right eye, ischemic CRVO; b: left eye, healthy. Abbreviations used: *CRAO* central retinal artery occlusion, *CRVO* central retinal vein occlusion.

Figure 1 shows the original OCT images of the five patients.

All 10 images of all five patients were presented to all examiners in a randomly changing sequence ten times. All the measurements were of usable quality. Every examiner marked areas in the designated retinal layers as described earlier to the best of his or her knowledge and experience. All graphic files were evaluated as described earlier, and the statistical data of the individual patients and the individual examiners were analyzed with the software described above.

## Results of the measurement system analysis

To assess the variation for each source of measurement error, we calculated the variance components and the percentage of contribution for each retinal layer. In Table 2, we present the results of the measurement system analysis (MSA), which partitions the total variation.

**Table 2:** Results of the measurement system analysis.

Gage *R*&*R*	VB	IMRL	MRL	IRL	ORL	VB	IMRL	MRL	IRL	ORL
	Percentage of contribution					Percentage of standard deviation				
Total Gage R&R	**1.11**	**0.94**	**4.94**	**2.56**	**3.77**	**10.55**	**9.71**	**22.22**	**15.99**	**19.41**
Repeatability	**0.79**	**0.62**	**2.80**	**1.42**	**2.60**	**8.91**	**7.86**	**16.74**	**11.90**	**16.14**
Reproducibility	**0.32**	**0.33**	**2.14**	**1.14**	**1.16**	**5.65**	**5.70**	**14.62**	**10.67**	**10.78**
Examiner	0.09	0.00	0.55	0.12	0.53	3.07	0.00	7.42	3.49	7.27
Eye: Examiner	0.22	0.33	1.59	1.02	0.63	4.74	5.70	12.60	10.09	7.96
Part-To-Part	**98.89**	**99.06**	**95.06**	**97.44**	**96.23**	**99.44**	**99.53**	**97.50**	**98.71**	**98.10**
Total Variation	100.00	100.00	100.00	100.00	100.00	100.00	100.00	100.00	100.00	100.00

Abbreviations used: *VB* vitreous body, *IMRL* innermost retinal layer, *MRL* middle retinal layer, *IRL* inner retinal layer, *ORL* outer retinal layer, *R&R* repeatability and reproducibility.

Ideally, only a very small part of the variance should be due to repeatability and reproducibility, which reflects the influence of the examiner (reproducibility) and the measurement method (repeatability). Differences between eyes (part-to-part) should account for most of the variance and show a reliable influence of the condition of the eye. The total Gage R&R is the sum of Repeatability and Reproducibility. For all the layers in our measurement system, the contribution from part-to-part (eye-to-eye) variation is high (> 95%), and the measurement system can reliably distinguish between parts (eyes). The analysis revealed that all the layers are useful parameters for the measurement system.

Overall, the measurement method showed good repeatability by the examiner and inter-examiner reproducibility for the retinal layers. The variations found for repeatability and reproducibility are not expected to be clinically relevant.

## Results of Lin’s concordance correlation coefficient and the percent study variation Gage R&R

The results of this statistical evaluation are divided into data for repeatability and reproducibility. The healthy eyes of all patients were used as controls, and the affected eyes were labeled diseased.

### Repeatability

Table 3 shows the results of Lin’s CCC (sorted) for repeatability and the total gage R&R of the percent study variation of the measurement for all examined eyes, healthy eyes and diseased eyes.

**Table 3:** Lin’s concordance correlation coefficient for repeatability and the total Gage R&R for all examined eyes, healthy samples and diseased samples.

Layer	All samples		Healthy samples		Diseased samples	
	Lin’s CCC	Gage *R*&*R*	Lin’s CCC	Gage *R*&*R*	Lin’s CCC	Gage *R*&*R*
	as %					
**VB**	99.12	8.91	94.13	21.85	99.32	7.42
**IMRL**	99.31	7.86	98.12	12.33	89.90	28.81
**MRL**	96.90	16.74	84.40	36.07	96.41	17.06
**IRL**	98.43	11.90	97.18	15.10	90.43	28.09
**ORL**	97.11	16.14	75.39	45.41	97.80	13.31

Abbreviations used: *VB* vitreous body, *IMRL* innermost retinal layer, *MRL* middle retinal layer, *ORL* outer retinal layer, *CCC* concordance correlation coefficient, *R&R* repeatability & reproducibility.

The repeatability of the measurements for all the samples revealed that Lin’s CCC was “substantial” or better (> 95%) for all the retinal layers. The IMRL (99.31%) and VB (99.12%) values were “excellent”; the IRL (98.43%), ORL (97.11%) and MRL (96.90%) values were “substantial” for repeatability. The Gage R&R reflects these findings, with values < 30% for IMRL (7.86%), VB (8.91%), IRL (11.9%), ORL (16.14%) and MRL (16.74%).

The IRL showed a “moderate to substantial” (> 90%) repeatability of the measurement in healthy (97.18%) and diseased (90.43) eyes. For IMRL, a “substantial” value in healthy (98.12%) eyes was calculated. For diseased eyes, the level was just missed at 89.90%, although Gage R&R indicated values below 30%. The repeatability of the measurements for the MRL (CCC 84.4%, Gage R&R 26.07%) and ORL (CCC 75.39%, Gage R&R 45.41%) in healthy eyes are not good enough to use these retinal layers for the measurement system.

### Reproducibility

**Fig. 2:**
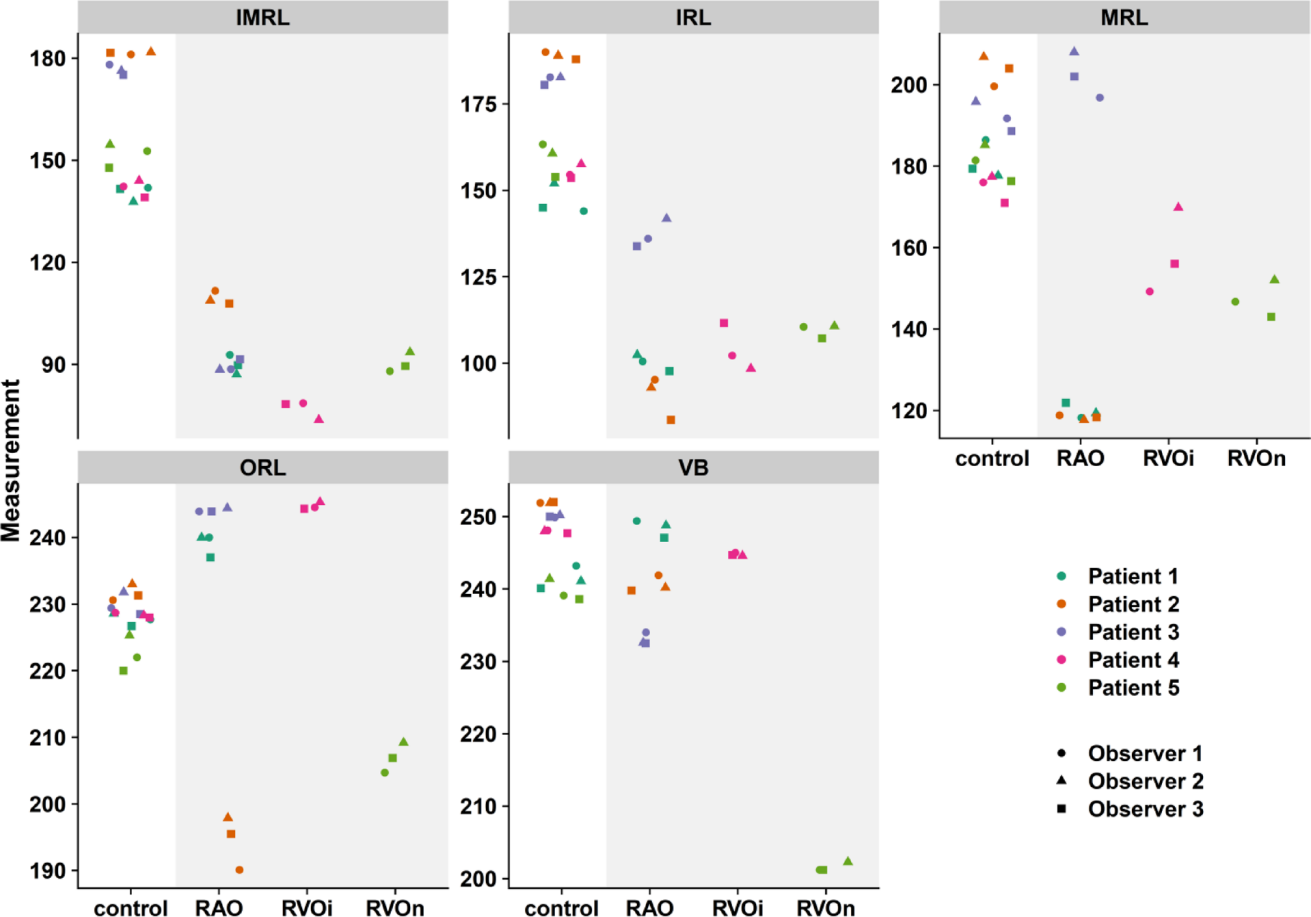
Mean measurements per observer. Abbreviations used: *VB* vitreous body, *IMRL* innermost retinal layer, *MRL* middle retinal layer, *ORL* outer retinal layer, *RAO* retinal artery occlusion, *RVO* retinal vein occlusion (*i* ischemic, *n*s non ischemic).

The dot plot in Fig. 2 shows the mean gray values of the measurements from the individual observer for the different conditions and for the separate layers.

As expected, there was no significant difference in the gray values of the VB between the diseased and control eyes, as the VB is not directly affected by vascular disease. The inner retinal layers (IMRL, IRL) show good discrimination between affected and unaffected eyes. The deeper the layer (MRL, ORL), the more difficult the discrimination becomes as the variance between the affected and control eyes increases.

Table 4 shows the results of the sorted Lin’s concordance correlation coefficient for reproducibility and the total gage R&R of the percent study variation for all examined eyes, healthy eyes and diseased eyes.

**Table 4:** Lin’s concordance correlation coefficient for reproducibility and the total Gage R&R for all examined eyes.

Layer	All samples		Healthy samples		Diseased samples	
	Lin’s CCC	Gage *R*&*R*	Lin’s CCC	Gage *R*&*R*	Lin’s CCC	Gage *R*&*R*
	as %					
**VB**	99.57	5.65	96.13	16.26	99.76	3.98
**IMRL**	99.58	5.70	98.37	10.98	91.70	25.41
**MRL**	97.33	14.62	87.22	29.35	96.87	15.45
**IRL**	98.59	10.67	96.63	16.40	94.67	19.23
**ORL**	98.45	10.78	80.90	35.30	98.99	8.30

Abbreviations used: *VB* vitreous body, *IMRL* innermost retinal layer, *MRL* middle retinal layer, *ORL* outer retinal layer, *CCC* concordance correlation coefficient, *R&R* repeatability & reproducibility.

When all the samples were examined, Lin’s CCC for reproducibility was “substantial” or better (> 95%) for all the retinal layers. In detail, the CCC is “excellent” for IMRL (99.58%) and VB (99.57%). “Substantial” values were shown for the IRL (98.59%), ORL (98.45%) and MRL (97.33%). This is confirmed by the Gage R&R values < 30% with IMRL (5.7%), VB (5.65%), IRL (10.67%), ORL (10.78%) and MRL (14.62%). In healthy and diseased eyes, there is a consistently “moderate to substantial” (> 90%) reproducibility of the measurement for the inner retinal layers (IMRL, IRL). In the diseased samples, however, this difference was greater, which we attributed to the large change in the retina in the case of vascular occlusion in comparison with the vitreous body.

The deeper the retinal layers are, the less reliable and reproducible the measurements are.

### Six Sigma Gage R&R for all samples referring to the individual retinal layers

**Fig. 3:**
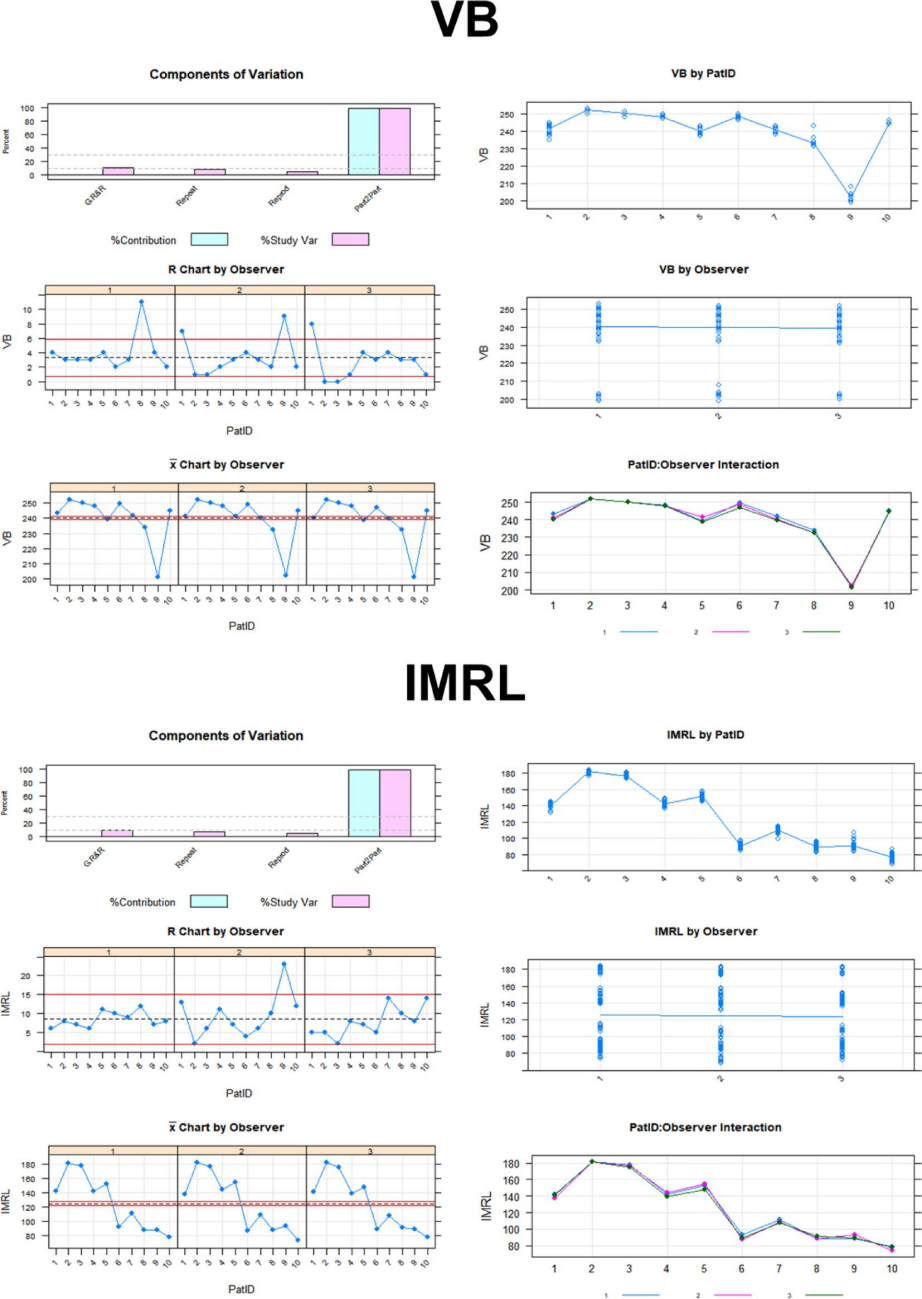

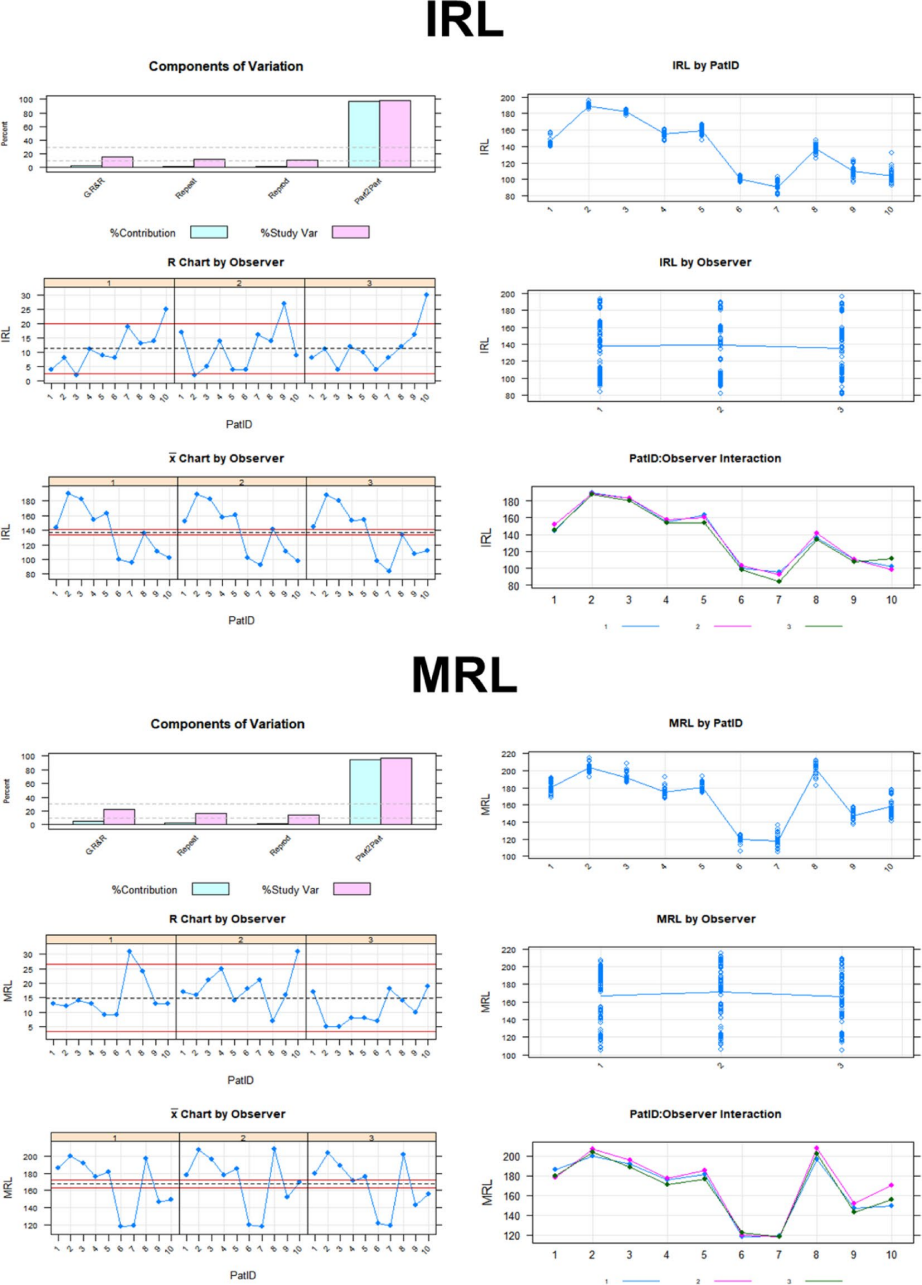

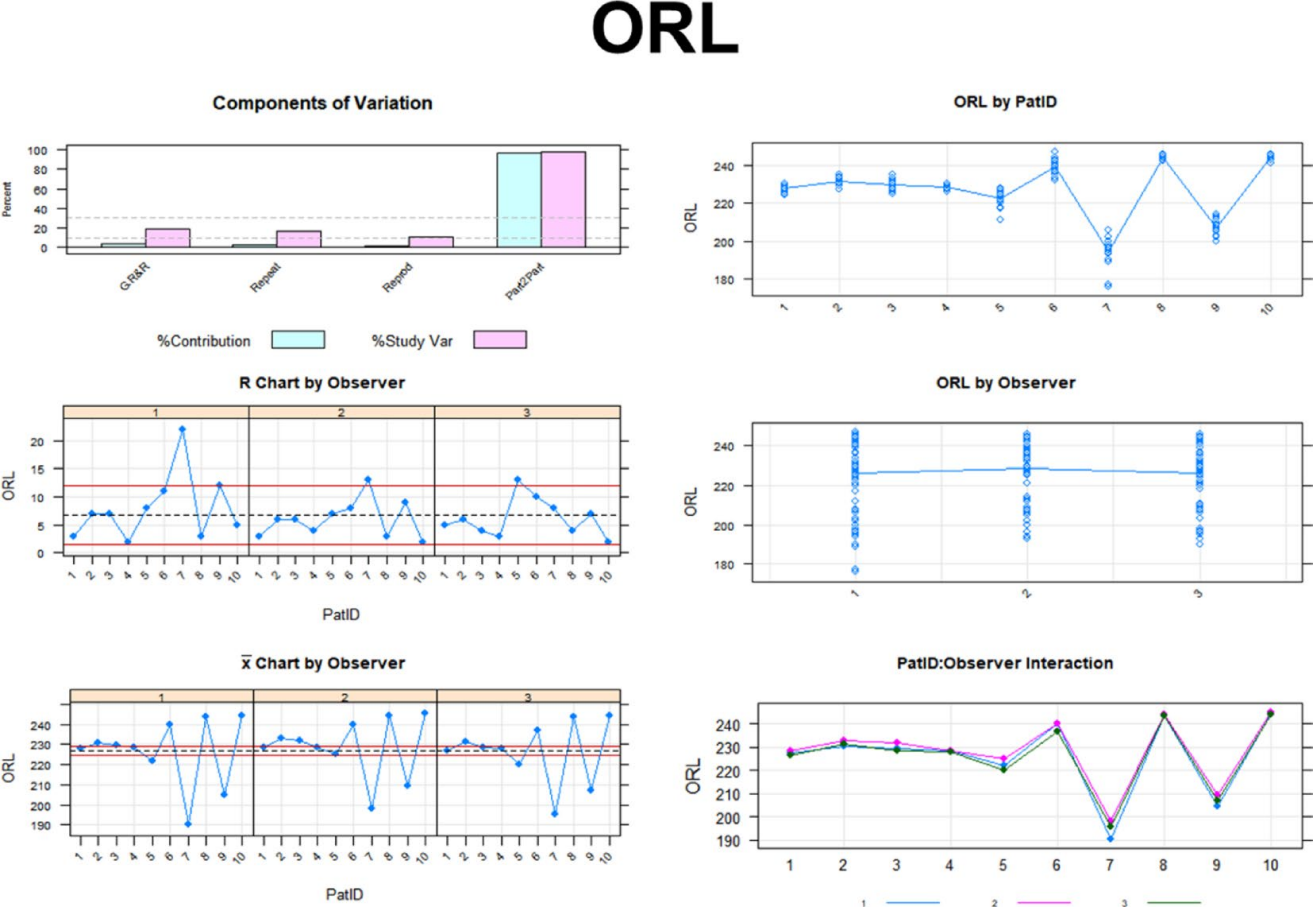
Detailed Gage R&R analysis for reproducibility and repeatability for all eyes related to the individual retinal layer.

Figure 3 shows the detailed Gage R&R analysis for reproducibility and repeatability for all eyes. This shows the influence of each of the conditions and examiners on the variance in the measurement system related to the individual retinal layer.

### Differences between the groups

**Fig. 4:**
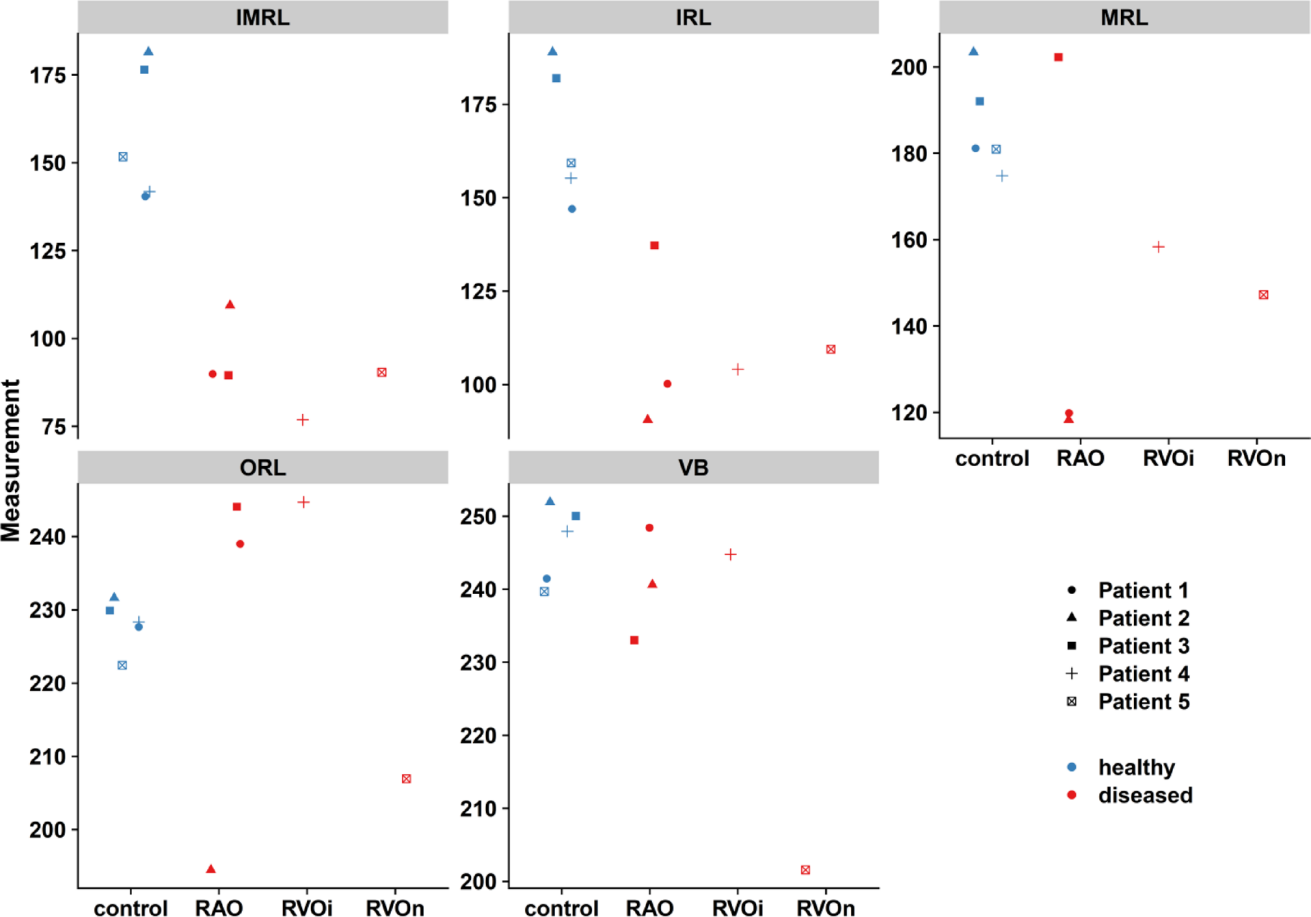
Mean measurements per condition. Differences between the groups (healthy = blue, diseased = red) are visualized via a dot plot with the means of the gray values for the individual layers. A separable distinction between diseased and healthy eyes is evident for the IRL and IMRL layers. Abbreviations used: *VB* vitreous body, *IMRL* innermost retinal layer, *MRL* middle retinal layer, *ORL* outer retinal layer, *ROA* retinal artery occlusion, *RVO* retinal vein occlusion (*i* ischemic, *n* non ischemic).

To investigate the differences between healthy and diseased eyes, the measurements of the different examiners were averaged, and the groups were compared. Figure 4 shows the mean values of the different conditions for the individual layers as a dot plot.

Owing to the small group size, the precondition of a normal distribution for the t test cannot be reliably assessed from the sample alone. Therefore, we additionally used the Mann‒Whitney U test to compare the values of the healthy and diseased eyes (Table 5). The t test showed a significant value for the IMRL and the IRL. The p value (*p* = 0.00794) for the U test was the same for both the IMRL and the IRL. This finding indicates that one group always has higher values for all patients than the other group does. This means that by measuring the gray values in the IMRL or IRL, it is possible to reliably distinguish between healthy and diseased eyes.

**Table 5:** P values of the Welch t test and Mann‒Whitney U test for comparisons of healthy and diseased eyes.

	*P* (t test)	*P* (U test)
IMRL	**0.00039**	**0.00794**
IRL	**0.00086**	**0.00794**
MRL	0.07083	0.09524
VB	0.21653	0.22222
ORL	0.84859	0.69048

## Discussion

Our group was able to describe gradual changes in the SD-OCT reflectivity of several retinal layers in the past. There are different forms and stages of changes depending on the underlying retinal problem. We were able to successfully classify the severity grades of retinal artery occlusions^9^ and demonstrate the similar nature of the changes in branch and central retinal artery occlusions^10^. Furthermore, we were able to show the effects of retinal vein occlusions on reflectivity^26^.

The drawback of all of the aforementioned works is that there is no objective way of obtaining the needed data. OCT data are subject to highly complex mathematics^46^ and, ultimately, data conversion and compression. Additionally, the selection of the OCT images, the acceptance of adequate image quality and, most importantly, the marking of the respective retinal layers’ representative zones on pathologically altered retinal layering are subject to individual examiners’ experience.

Hence, it is mandatory to be able to – best – know or – at least – estimate the amount by which those factors influence the measurements. Although OCT raw data are highly modified and logarithmically compressed,^46^ these algorithms might account for systematic errors. The individual errors of examiners are not systematic. Therefore, they may considerably alter the data, and hence, in this paper, we focused on them.

Our approach has been guided by the following assumptions:

By selecting individual patients’ affected and healthy eyes, we were able to obtain the best matched control images. By selecting one patient from among every up-to-date evaluated condition (ischemic and non-ischemic retinal vein occlusion and incomplete, subtotal and total retinal artery occlusion), we covered all possible appearances and conditions reviewed here and therefore covered the whole spectrum of changes in retinal reflectivity from greatest (total central retinal artery occlusion) to least (non-ischemic central retinal vein occlusion) to healthy. By selecting experienced and unexperienced examiners, we were able to estimate the influence of experience. Finally, by keeping the instructions of the unexperienced to a minimum (individual explanations were provided only for the use of the imaging software, whereas the conditions for selecting the retinal layers’ areas were left to already published papers), the quality of the published instructions for reproducing our data independently of our group was also tested.

By using a measurement system analysis, we were able to pinpoint the repeatability variance of the measurements to 0.79% for the VB, 0.62% for the IMRL, 2.80% for the MRL, 1.42% for the IRL And 2.60% for the ORL. The reproducibility variance was 0.32% for the VB, 0.33% for the IMRL, 2.14% for the MRL, 1.14% for the IRL and 1.16% for the ORL. Our published method of measuring retinal reflectivity on the basis of OCT images can be safely applied to all retinal layers, although the reflectivity measurements become less reliable for deeper retinal layers. For all the examined groupings, the reproducibility of the inner retinal layers (IMRL, IRL) and vitreous body were at least moderate (> 90–95%), in most cases substantial (> 95–99%) or even excellent (> 99%). The deeper the retinal layers are, the less reliable and reproducible the measurements are, with MRL and ORL being sufficiently useable only under certain conditions.

These findings are supported by the results of Lin’s CCC. For the VB, IMRL and IRL, the results are substantial to excellent in terms of repeatability and reproducibility. This was observed for all the eyes, all the healthy eyes and all the diseased eyes. Only the deeper retinal layers MRL and ORL cannot be reliably measured in groups of healthy eyes or diseased eyes.

These data support the continuous use of our method for evaluating retinal reflectivity and, consequently, retinal ischemia for more detailed measurements in the future.

Limitations to our study are as follows:

We included only one patient from each category. This approach covers the whole spectrum of variation over all the pathologies, but the use of at least two covering the whole spectrum of values in each of the categories could provide further insight into the linearity of the method. Only one experienced examiner contributed to the study. More experienced examiners might refine the data. As noted before, we had to rely on heavily modified data from the OCT images and were not able to double check our results against the raw data. Although we consider this to be a systematic error, it might contribute to misinterpretation.

## Summary

Manual measurement of the reflectivity of retinal layers obtained from SD-OCT images is reliable, repeatable and reproducible, and reflectivity can be safely used as a quantitative parameter for further workup.

Although experience is helpful, even unexperienced observers are able to produce reliable and reproducible values.

The differences in retinal layer reflectivity measurements are mainly accounted for by the differences in the conditions of the eyes themselves (healthy or diseased, with respect to the disease condition and grade of severity) and are not due to data evaluation or inter- or intra-observer variations.

However, both reliability and reproducibility decrease in deeper retinal layers, making the inner retinal layers the most promising ones for future work.

Our previously described method can be safely used for statistical evaluation or intra-patient comparisons for timelines or effects of possible therapies.

## Methods

The present study adhered to the tenets of the Declaration of Helsinki. The study was approved by the Institutional Review Board of Technische Universität Dresden (Dresden, Germany). Because of the retrospective design and because there were no study-related investigations, informed patient consent was waived by the Institutional Review Board of Technische Universität Dresden (Dresden, Germany). The investigation has been registered in ClinicalTrials.Gov (ClinicalTrials.gov Identifier NCT03061526).

### Patient selection

The patient database from Dresden University Eye Hospital was reviewed for billing codes of retinal vessel occlusions according to the International Classification of Diseases, 10th Revision, between September 2011 and December 2021. As previously published^9^the included patients had to meet the following criteria: First, they had to be diagnosed with acute central retinal vein occlusion (CRVO; ischemic or non-ischemic), acute central retinal artery occlusion (CRAO) or branch retinal artery occlusion (BRAO). BRAO was defined as an occlusion of one of the branches of the central retinal artery, which should have affected at least in part paramacular regions central of the retinal vessel arcades, but less than half of the retina. Second, there had to be vision loss or a defect in the visual field occurring and diagnosed within 7 days of the initial visit. Third, the patient must have had SD-OCT at the initial visit. Fourth, the OCT image quality score of said SD-OCT had to be > 30. Patients with a history of the following were excluded: ocular trauma or presence of macular disease, severe nonproliferative or proliferative diabetic retinopathy, other retinal vascular diseases, glaucoma, myopic retinopathy, or other diseases interfering with OCT images in any one of the eyes (e.g., vitreomacular traction, epiretinal membrane) as well as one-eyed patients. The included patients then underwent ophthalmic examinations, including fluorescein angiography and OCT, with previously described parameters^9^. Out of this large cohort, one patient was selected randomly from each of the following conditions: ischemic central retinal vein occlusion, non-ischemic central retinal vein occlusion, incomplete central retinal artery occlusion, subtotal central retinal artery occlusion, and total central retinal artery occlusion. Healthy contralateral eyes were used as controls so that every patient was represented by two OCT images obtained under conditions previously described^9,10,26^.

Importantly, the patients were mainly selected to represent the full range of changes in retinal reflectivity, from the most severe – seen in the previous studies in total central retinal artery occlusion^9^ – to least severe (non-ischemic central retinal vein occlusion)^26^ to healthy eyes^9,10,26^. The goal of this investigation was neither to establish the pathologic status of the eyes nor to prove that previously used grading systems are true or wrong but rather to assess the method as a proof of principle on different levels of retinal reflectivity, which, for obvious reasons, would not be found in healthy eyes only. However, for further statistical analysis, the eyes were grouped again according to the disease.

### Definition of retinal layers and reflectivity measurements

For the nomenclature of the analyzed retinal layers and the abbreviations used, please see Table 6.

**Table 6:** Nomenclature of analyzed layers in retinal vessel occlusion and contralateral healthy eyes.

**Vitreous body (VB)**		
Ganglion cell layer (GC)	**Innermost retinal layer** **(IMRL)**	**Inner retinal layer** **(IRL)**
Inner plexiform layer (IPL)		
Inner nuclear layer (INL)	**Middle retinal layer** **(MRL)**	
Outer plexiform layer (OPL)		
Outer nuclear layer (ONL)	**Outer retinal layer** **(ORL)**	
External limiting membrane (ELM)		
Ellipsoid layer		
Retinal pigment epithelium (RPE)		

Abbreviations and definitions of layers used: *VB* vitreous body, *IMRL* the innermost retinal layer, including the retinal nerve fiber layer, ganglion cell layer and inner plexiform layer, *MRL* middle retinal layer, including the inner nuclear layer and the outer plexiform layer, *ORL* the outer retinal layer, including the structures from the inner border of the outer nuclear layer to the outer border of the retinal pigment epithelium.

The detailed method for measuring the reflectivity of separate retinal layers has been described previously for retinal artery occlusions^9,10,27^.

Although the underlying pathology, timeline, established classification, therapeutic approaches, prognosis, etc., differ substantially between arterial and venous retinal occlusion, the same approach of measuring retinal layer reflectivity has been successfully used for retinal vein occlusions^26,28^.

### Randomization of images, repeatability, reproducibility and instructions

The images of the RVO affected eye and the control eye of each patient were randomly presented to each of the examiners (E.M., P.E., K.S.). The respective images were presented to each examiner ten times at different time points in a randomly changing sequence. The value of the gray level was read out and documented for the individual areas of the image, as already described.

The handling of the software was explained to the unexperienced investigators (P.E., K.S.) by an experienced investigator (E.M.). The criteria for selecting areas of interest were only given in the same way as those described in the past^9^.

### Statistical analysis

#### Overview

This work aims to prove and statistically support a new approach for evaluating OCT images: its goal is to establish a new measurement method. Therefore, measurement system analysis (MSA) is a suitable statistical approach. MSA basically consists of estimating two different stability measures: Repeatability (intra-observer; variability in measurements when the same examiner measures the same part multiple times) and reproducibility (inter-observer; variability in measurements when different examiners measure the same part). They were quantified via the two complementary methods Total Gage Repeatability & Reproducibility (Total Gage R&R) and Lin’s concordance correlation coefficient (Lin’s CCC). The measurement dataset included data from 3 observers, each of whom measured 10 eyes from 5 patients. Each eye was measured 10 times per observer.

#### Gage Repeatability & Reproducibility (Gage R&R)

Gage R&R is a statistical method for measurement system analysis (MSA) and has been well covered in the literature^29–39^. It quantifies the amount of variation in the measurement data due to the measurement system. By comparing the measurement variation to the total variability observed, the capability of the measurement system can be defined. Measurement variation consists of two important factors: repeatability (variation due to equipment) and reproducibility (variation due to inspector or operator). In this work, Gage R&R was calculated via the analysis of variance (ANOVA) method with the two factors eye and observer and their interaction. The total Gage R&R shows how much of the measurement variation can be attributed to a lack of repeatability and a lack of reproducibility. The counterpart is part-to-part variation, which reflects the degree to which the differences measured reflect differences in the variables. The total measurement system variation (which is not listed here) is the sum of the variation in the total gauge R&R and part-to-part variation (the variability of the individual data).

For measurement system analysis, a total Gage R&R value of Less than 10% of the total variation indicates that the precision of the measuring system is high enough to measure the investigated quantity appropriately. This would be reflected by a part-to-part value of > 90%. Tolerance values of 10–30% (or, part-to-part of 70–90%) are acceptable, depending on the measuring system, And values above 30% (part-to-part of < 70%) indicate that the measuring system is not suitable.

#### Lin’s concordance correlation coefficient (CCC)

Lin’s concordance correlation coefficient (CCC) measures the degree of agreement and correlation between two sets of continuous data^40–44^. It ranges from 0 to 1 and is often expressed as a percentage.

For Lin’s concordance correlation coefficient (Lin’s CCC), the following grading was used: less than 90% was considered poor, 90–95% was considered moderate, 95–99% was considered substantial and > 99% was considered excellent^45^.

#### Data processing and software used

Data for continuous variables are expressed as the mean ± standard deviation (SD). To determine statistical significance, *p* < 0.05 was chosen.

The software used for image acquisition, interconversion between “black-on-white” and “white-on-black” OCT scans, export and analysis have also been described previously^9^. In addition, the statistical software R (www.r-project.org, version 4.2) and SPSS were used for statistical analysis. In particular, the R package SixSigma was used for the Gage R&R analysis.

## Data Availability

The authors report that the datasets used and/or analyzed during the current study are available from the corresponding author upon reasonable request.
